# Insights to optimizing health extension workers’ time use and efficiency in Ethiopia: a qualitative study

**DOI:** 10.1080/16549716.2025.2548629

**Published:** 2025-08-28

**Authors:** Kora Tushune Godana, Yibeltal Kiflie Alemayehu, Negalign Berhanu Bayou, Yohannes Kebede, Peter Decat

**Affiliations:** a Department of Health Policy and Management, Public Health Faculty, Jimma University, Jimma, Ethiopia; b Research, Monitoring and Evaluation, MERQ Consultancy PLC, Addis Ababa, Ethiopia; c Department of Health, Behavior and Society, Faculty of Public Health, Jimma University, Jimma, Ethiopia; d Department of Public Health and Primary Care, Faculty of Medicine and Health Sciences, University of Ghent, Ghent, Belgium

**Keywords:** Health extension program, health extension workers, efficiency, time use, Ethiopia

## Abstract

**Background:**

The Health Extension Program (HEP) is Ethiopia’s flagship program introduced in 2003 to ensure equitable access to primary healthcare services. Recently, inefficiency in the time use patterns of staff of the HEP, including absenteeism and non-productive engagements, has been reported as a major challenge.

**Objective:**

This qualitative study explored what factors influenced their time use and efficiency.

**Methods:**

This is a qualitative study that was conducted in rural health posts (HPs) across Ethiopia in three rounds over a period of 12 months (17 May 2023 to 16 May 2024). Qualitative data were collected through key informant interviews (KIIs), in-depth interviews (IDIs), and focus group discussions (FGDs) involving HEWs and other staffs of health posts, their supervisors, Kebele leaders, Women’s Development Army (WDA), teachers, and other community members. A total of 52 KIIs, 31 IDIs, and 28 FGDs were conducted in three rounds. Data were analyzed using ATLAS.ti 7.1.18 software.

**Results:**

HEWs commonly spent their time on productive non health or non-HEP activities such as those related to community-based health insurance, agricultural and rural development and education sectors, and other political assignments. It was also noted that HEWs could be available in their workplace, but might not be productive. Factors for the inefficiencies across HPs often pertained to environmental and demographic features, community trust and engagement, local administration; human resource development and management practices, multisectoral collaboration; and underlying causes of absenteeism including motivation.

**Conclusion:**

Inefficiencies are common among HEWs. Strategies need to be devised focusing on the identified modifiable factors such as improving accountability and performance management practices, introducing incentive mechanisms to keep HEWs motivated, improving accessibility to transportation services, and security conditions, workforce to population size ratio; and capacity building.

## PAPER CONTEXT

● **Main findings:** Health Extension Workers (HEWs) commonly spent their time on productive activities which are not related to Health Extension Program (HEP) packages. Absenteeism or late job attendance was also common, and sometimes, they could be available at workplace, but might not be productive. Factors for the inefficiencies often pertained to the community context, health system leadership and governance practices, and the underlying causes of absenteeism such as long travel time to from home to workplace, inaccessible transportation services, weak security conditions, lack of motivation, and household and social responsibilities of HEWs who are mostly female.

● **Added knowledge:** Although there is some understanding on inefficient time use patterns of HEWs, including absenteeism and non-productive engagements, this paper fills knowledge gap about the factors that influence their time use and efficiency.

● **Global health impact for policy action:** Interventions focusing on the identified modifiable factors such as improving accessibility to transportation services, security conditions, and introducing incentive mechanisms to keep HEWs motivated are required for the success of HEP implementation.

## Background

Ethiopia faces a triple burden of diseases with continued high rates of communicable diseases and an increasing burden of non-communicable diseases (NCDs) and injuries, resulting in millions of Ethiopians suffering from diseases for which well-proven, cost-effective interventions exist [1]. As of 2021/2022, the top five reasons for outpatient department visits included pneumonia, acute respiratory infections, dyspepsia, functional intestinal disorders, and typhoid and paratyphoid illnesses [2]. NCDs account for 42% of deaths in the country [3]. Risk factors driving morbidity and mortality are largely linked to unhealthy behaviors that could be addressed with appropriate health promotion and disease prevention activities [1].

To address the pressing challenges of access to equitable health services, the Government of Ethiopia has designed and implemented a health policy that considers the major causes of morbidity and mortality [4]. As a key strategy, the country introduced the Health Extension Program (HEP) in 2003 – an innovative community health program that institutionalized the formerly fragmented village health services into a comprehensive package of primary healthcare delivered by government salaried Health Extension Workers (HEWs) [5]. The HEP is a community-based strategy to provide health promotion, disease prevention, and selected curative health services at the community level, free of charge. The underlying philosophy of HEP is that ‘if the right knowledge and skills are transferred to households, then they can take responsibility for producing and maintaining their health and society at large.’ [6]. Currently, the program involves more than 42,000 HEWs and more than 18,000 health posts (HPs) [2] to implement 18 service packages in four areas: 1) hygiene and environmental sanitation, 2) disease prevention and control, 3) family health, and 4) health education and promotion [5].

To contribute to the realization of universal health coverage by 2035, the country has developed a roadmap to optimize the HEP that includes six interrelated strategic objectives: ensuring equitable access to essential health services, improving the quality of health services, ensuring sustainable financing, strengthening community engagement and empowerment, and ensuring resilience by maintaining the provision of essential services during emergencies [7]. The roadmap intends to ensure equitable access to essential health services by expanding HEP service packages to align interventions with changes in disease epidemiology, and to meet the needs and expectations of communities. Similarly, the quality of HEP services will be improved through introducing changes in HEP workforce, supplies management, infrastructure and basic amenities, service delivery processes, and governance and leadership. Depending on the category of HEP implementation platform (i.e. basic HPs, comprehensive HPs, and HEP units in health centers (HCs)) and setting (urban or rural), staffing for HEP will involve multidisciplinary team composed of male and female health officers, nurses/midwives, environmental health professionals, and HEWs. The plan is to double the number of health professionals working at HP level by 2030 [7].

Despite the significant achievements of HEP in the past few decades, e.g. in reducing under-five mortality rates by 67% [5], HEP is facing critical challenges [6,8] such as high turnover rates of HP staff [6], low quality of services [6,8,9], limited capacity of HEWs [8,10,11], absence from work place [12], and lack of motivation [8]. The available literature demonstrated inefficiency of community health workers mostly using quantitative methods [12–19]. Only three of these studies [12,17,19] determined how the Ethiopian HEWs allocate their time, and two studies [18,19] explored their time use qualitatively, including barriers and facilitators for effective time use. For instance, Tilahun et al. [17] found that only a minority of HEW time is spent on providing health education and services, and substantial time is spent waiting for clients. Another study similarly noted that over the course of a week, HEWs divided their time between the HP (51%) and the community (37%), with the remaining 11% spent elsewhere [12]. However, evidence on the reasons for inefficiency are generally scarce. Therefore, this study explored how HEWs spent their time across various tasks, and what factors influenced their time use and efficiency. An in-depth understanding of these questions will help to identify opportunities to improve the efficiency and effectiveness of HEWs.

## Methods

### Study setting

The study was conducted in rural HPs across Ethiopia. As satellites of Primary Health Care Units (PHCUs), rural HPs are established in every *Kebele*—the lowest administrative unit of the Ethiopian Government – and are designed to serve a catchment population of approximately 5,000 individuals in rural areas. The recent HEP restructuring initiative categorized rural HPs into three types: comprehensive HPs, basic HPs, and HEP unit in HCs. Basic HPs are typically staffed by two female HEWs who provide HEP packages. In contrast, merged HPs are upgraded to include the two HEWs and nurses – HEWs who have pursued advanced career education – with the aim of strengthening outreach and preventive activities linked to HCs. Comprehensive HPs are designed to provide clinical and childbirth services that are not available in the basic and merged HPs; and staffed by a wider range of health professionals, including health officers, environmental health experts, pharmacists, and midwives, in addition to the two HEWs [7]. The study included 15 HPs across these categories, representing seven agrarian regions (Sidama, Central Ethiopia, Southern Ethiopia, South Western Ethiopia, Oromia, Amhara, and Harari), and two pastoralist regions (Afar and Somali).

### Study design

Using case study approach to qualitative study, this study aimed to understand HEWs’ time use patterns and the drivers of efficiency and inefficiency in their time use patterns in the Ethiopian HEP context. The study was conducted in three rounds over 18 months to see seasonal variations in time use. We analyzed HEWs’ daily, weekly, and monthly activities to assess work efficiency and identify challenges. The study explored how HEWs allocated their time, the extent to which they followed planned tasks, the impact of unplanned and non-HEP activities on HEWs’ efficiency, and the factors influencing time use.

### Study population

This study was conducted in three rounds over 12-month periods, between 17 May 2023, and 16 May 2024. Key stakeholders involved in the implementation and management of the HEP strategy were purposively selected for the study. The purposive selection was aimed to achieve symbolic representation and capture the diversity of perspectives across a broad range of stakeholders. These included beneficiaries, community structures, collaborative sectors, service providers, supervisors, program coordinators, and health system leaders. Specifically, the participants included HEWs, their supervisors, *Kebele* leaders, Women’s Development Army (WDA) members and leaders, teachers, and community members including resident women and men, and female and male youths. The selection ensured representation across diverse geographic and terrain conditions, livelihood contexts (pastoralist and agrarian), types of HPs, and the range of health workers involved in service delivery.

### Data collection methods and tools

In each round of data collection, the participants were involved in focus group discussions (FGDs), in-depth interviews (IDIs), or key informant interviews (KIIs). FGDs were organized to capture a broad range of experiences and normative perspectives on time use from diverse participant groups. KIIs were conducted with strategically positioned individuals, including program coordinators, supervisors, and leaders from both the health system and the community. IDIs involved individuals with extensive experience and rich, context-specific knowledge. Sampling was guided by saturation of ideas, and each FGD included 6–10 participants. A total of 52 KIIs, 31 IDIs, and 28 FGDs were conducted (Table 1).

**Table 1.** Distribution of participants by region and representation.

Characteristics	KII (*n* = 52)	IDI (*n* = 31)	FGD (*n* = 28)
	Number (%)	Number (%)	Number (%)
**Region**			
Afar	8(15.4)	4(12.9)	7(25.0)
Amhara	3(5.8)	0	0
Oromia	9(17.3)	5(16.1)	6(21.4)
Sidama	10(19.2)	4(12.9)	0
Central Ethiopia	10(19.2)	4(12.9)	0
Harari	3(5.8)	5(16.1)	4(14.3)
Somali	3(5.8)	3(9.7)	6(21.4)
South Ethiopia	3(5.8)	3(9.7)	0
Southwest Ethiopia	3(5.8)	3(9.7)	5(17.9)
**Category of participants**			
**KII**			
HEWs	25(48.1)		
HEP supervisor (HC head)	14(26.9)		
HEP coordinator (District Health Office head)	13(25.0)		
**IDI**			
WDA leader		7(22.6)	
Women’s Affairs Office head		6(19.4)	
Teacher		6(19.4)	
Kebele administrator/manager		12(38.7)	
**FGD**			
Female youth			5(17.9)
Male youth			4(14.3)
Resident women			4(14.3)
Household heads (male)			4(14.3)
WDA leaders			5(17.9)
Community leaders, e.g. religious leaders			6(21.4)

Customized guides were used for FGDs, IDIs, and KIIs which included open-ended questions on factors influencing HEWs’ time use, motivations, challenges, and community expectations. Key questions focused on time use, variations in time use, and factors affecting HEP efficiency with further probing as needed. Data collection tools were adjusted between rounds to ensure comprehensive coverage of relevant topics. Bachelor’s and master’s degree holders with extensive qualitative interviewing and local language skills conducted face-to-face interviews at respondents’ respective natural settings: office, HPs, *Kebele*, and community residential villages to ensure participants’ comfort and openness. Informed consent was obtained from each respondent before interview. FGDs were facilitated by a moderator and a note-taker, while IDIs and KIIs were conducted one-on-one in a conversational style. The interviews lasted 1:00–1:45 hours, and transcribed verbatim. All sessions were audio-recorded with permission, and extensive field notes were taken to capture non-verbal cues and contextual observations. At the end of each day, debriefing sessions were held among data collectors to reflect on emerging themes, address challenges, and adjust probing techniques as needed. Data collection continued until saturation was reached across participant categories and study sites.

### Data analysis

Data were managed using ATLAS.ti 7.1.18 Software and analyzed using thematic analysis method. Two PhD-level qualitative researchers independently read the transcriptions and developed a codebook from the text. Four coders then applied the codebook, with emerging codes discussed and added as needed. The final code structure was approved by the lead coders. Texts on time use and influencing factors were interpreted using open coding, and themes and sub-themes were generated by clustering codes. For the interpretation of the data, the analysis primarily focused on how HEP staff allocated their time – specifically, how time was spent on HEP versus non-HEP activities – and the factors influencing these patterns of time use across different tasks and contexts. We reported the results using the consolidated criteria for reporting qualitative research (COREQ). A completed COREQ checklist is provided in Online Supplementary File 1.

### Rigor

Credibility was ensured by involving experienced teams in interviews, coding, and writing. Thick descriptions were provided for the themes and sub-themes, supported by quotations that added value to the interpretations. Lead analysts, who were qualitative research and health system experts, maintained subjective neutrality to minimize interpretation bias. Peer debriefing and daily team interactions during interviews and coding further supported credibility. The diversity of HPs, settings, and participants enhanced the transferability of the findings. Saturation was evident in the clarity of descriptions and interactions across themes. The adequacy of the findings was confirmed by audits of internal and external evidence related to the HEP.

### Ethical considerations

The study received ethical approval from Institutional Review Board of the Ethiopian Public Health Association (Reference: EPHA/06/586/23). Standard ethical procedures were followed, including training data collectors and supervisors in research ethics, seeking informed consent, and ensuring participant privacy and confidentiality. Written informed consent was obtained from all participants. Participation was voluntary, and all data were confidentially managed by the research team and stored for five years in a secured database owned by MERQ Consultancy PLC. The report was de-identified to protect participants’ confidentiality.

## Results

### Characteristics of participants

Participants included a diverse group of individuals such as HEWs, other health professionals involved in the HEP (e.g. nurses and HC heads), *Kebele* leaders, community leaders, youths, and women network leaders. They were drawn from various settings, including agrarian and pastoralist, and different types of HPs. The participants’ ages ranged from 26 to 80 years, and their educational background varied from no formal schooling to a BSc degree. Their professional and volunteer experience ranged from 7 to 20 years. The participants held various positions, including leading WDA, HC, HP, and *Kebele* Administration. The distribution of participants by region and representation is summarized in Table 1.

### Data structure

The analysis generated five themes to characterize HEWs’ time allocation and use patterns, and three themes as key factors that influence time use. Each of the themes had various emergent themes/sub-themes (Table 2). The detailed results are provided subsequently.

**Table 2.** Themes and sub-themes generated for analysis of time allocation and use patterns, and factors influencing time use in Ethiopia, 2023.

Themes	Emergent themes/Sub-themes
**Time allocation and use patterns**	
Number of work days and reported workload	• Typical work days • Busy work days • Less work busy • Atypical work days
Time spent on implementing HEP packages	● Facility-based services • Outreach services • Campaign-based services • Home visit/door-to-door services
Time spent on HEP-related administrative activities	● Documentation • Preparing performance reports
Time spent on HEP-related travel	• Distance from home to HP • Road access • Availability of transport services
Time spent on other activities*	● Collecting community-based health insurance (CBHI) premium • Serving guest visitors • Political meetings and support to other sectors on matters that are not directly related to HEP tasks, e.g. tax collection
**Factors that influence work efficiency and time use**	
Community context	● Environmental and demographic attributes • Community trust and engagement • Local administration
Health system leadership and governance	● HR development and management • Multisectoral collaboration • Work environment
HEWs’ job attendance	● Gender intersectionality • Long travel time from home to workplace • Limited access to transportation service • Lack of motivation

*All activities which were not included in the other themes were represented as “Other activities”.

## HEWs’ time allocation and use patterns

### Number of work days and reported workload

On typical days, HEWs mostly complied with their work plan and standard daily working hours, i.e. 8:30 AM to 5:30 PM, from Monday through Friday. Busy days of the week varied across HPs. Typically, HEWs consider Monday and Thursday as the busiest days of the week. Monday tended to be busy as it immediately followed the weekend, while Thursday was often a non-market day designated for campaigns or outreach activities. To cope with these demands, HEWs employed strategies such as prioritizing tasks, working for extra hours, and arriving to workplace as early as possible to avoid delays due to transportation issues if living far away from the HP.

Tuesday and Friday, days following heavy workloads, were commonly mentioned as less busy for HEWs. Facility-based service days at HPs were also considered less busy, particularly when adequate staff were available and reporting deadlines were not imminent. On atypical work days, HEWs often managed unplanned tasks such as supervisory visits from the Woreda Health Office or HC, visits by other guests or researchers, emergency events in the community, and other political assignments.

### Time spent on implementing HEP packages

HEWs spent a substantial portion of their time providing outreach and facility-based services, both preventive and curative. At the HP level, they particularly provided the following services two days a week: antenatal care (ANC), child immunization, family planning (FP) and contraceptive services, child nutrition, rapid malaria diagnosis and treatment, and management of diarrhea with oral rehydration salt and Zinc, all accompanied by health education. HEWs also spent 3 days per week providing outreach services: 1 day on campaigns and the other 2 days visiting homes.

Campaign-based and door-to-door services were time-consuming, particularly in areas with scattered settlements and rugged topographies, requiring longer travel time. During campaigns, they carried out a wide range of activities, including but not limited to: immunizations (both for children and pregnant women); child growth monitoring and/or malnutrition management; detection and referral of sick or severely malnourished children; vitamin A supplementation; identifying missed service appointments both for children and pregnant mothers; providing first aid; and health education on various topics, such as ANC, institutional delivery, nutrition, and sanitation and hygiene. These services were mostly provided at designated outreach sites, typically near schools. The HEWs were expected to cover all campaign sites within the Kebele, usually three to four sites every week ensuring that each site is reached monthly, or 12 times per year. In pastoralist settings, however, outreach services were limited due to unfavorable environmental conditions and widely dispersed households (HHs), as illustrated below:

The area where we live is far from the health post, so the health professionals don’t want to come to this area, so we have never received door-to-door services. (P7, Women’s FGD, Afar Region)

During the two-day home visit per week, HEWs primarily focused on the importance of the construction and use of latrine facilities and the safe disposal of solid and liquid wastes. They also commonly identified and referred pregnant women and sick or malnourished children for facility-based care provided first aid and delivered cross-cutting health education. Particularly, they spent a considerable amount of time providing health education on topics related to hygiene and environmental sanitation (HES) and family health components. The most common HES-related topics covered were latrine utilization, water safety, and health risks of living together with animals, and the need for separation. Similarly, health education sessions mainly focused on the following family health services: FP, ANC, nutrition, birth preparedness, newborn care, exclusive breastfeeding, and complementary feeding. The sessions targeted diverse audiences using multiple methods, such as health talks during home visits, discussions with couples, conversations with social networks, and school-based education. Participants recognized the critical role of health education in promoting health awareness and ensuring the adoption of healthy behaviors and uptake of recommended practices.

Health education is a cross-cutting strategy for HEP implementation. We have two approaches that are coordinated by HEWs: the one-to-five women’s network where one HEW trains five women in the community, and the one-to-thirty women’s network. Both meetings take place every 15 days to discuss various health topics and practices. Finally, they report these accomplishments to us. So, this demands much of their time. (KII, HC Head, Afar Region)

As part of the HEP implementation strategy, community engagement activities also required a significant amount of HEWs’ time. These activities included mobilizing key actors through engaging community-based structures and organizations such as the WDA, *Iddir*, and schools to support HEP activities. *Iddir* is a social institution used for mutual aid granting cooperative insurance within a specific community. HEWs also created model families to resonate best practices across the Kebele. Families were considered models when they demonstrated effective implementation of most of the HEP packages, for example, using locally available materials to construct latrine facilities that are properly used.

HEWs spend most of their time on tasks which are practiced at the community level, e.g., modeling of the Kebele or families. Modeling the Kebele requires accomplishing more activities and effort, and hence, takes more time. Particularly, activities related to the HES package should be implemented as this is the main criterion for Kebele modeling, which is very challenging. (KII, HC Head, Meskan, Gurage Zone)

### Time spent on HEP-related administrative activities

Administrative duties related to HEP took up a significant portion of the HEWs’ time. These mainly involved documentation and reporting activities. For instance, HEWs were busy updating family folders and registries of the 18 HEP packages. Besides, manual record-keeping and reporting processes often consumed much of HEWs’ time including their off-days. Some reporting forms should be completed in English, challenging less proficient HEWs.

There are gaps in reporting all these activities on weekly, monthly, quarterly, semi-annual, and annual bases. This partly relates to the reporting system of the program which requires completing all the reports in English, while majority of the HEWs are not proficient in the language. (KII, HC Head, Gurage Zone)

Sometimes, HEWs needed support from other health professionals to complete reports for which they emphasized the need for digitization of recording and reporting formats, and in local languages. Meetings with Kebele leaders and other sectors for HEP tasks also contributed to their administrative workload.

### Time spent on HEP-related travel

Long travel time reportedly contributed to a significant portion of the HEWs’ workday which varied by the geographic location of their workplace. In remote HPs without residential facilities for HEWs, travel time could take up a substantial number of hours due to poor road infrastructure, lack of transport services, and dispersed populations. These travel constraints directly impacted the efficiency of HEWs, often reducing the time available for direct facility or outreach-based activities.

HPs located far from the nearest HC often have a residence for HEWs to enable them to use their time effectively. In contrast, HPs close to the nearest HC often lack such a facility and HEWs should travel every day, competing for their productive time. (KII, HEP Supervisor, East Showa, Oromia Region)

… their workplace is in rural areas but they reside in [Sodo] town. They arrive at their workplace late and leave before the expected exit time, due to a shortage of transportation service. Long travel time is a common challenge for HEWs’ efficiency. (KII, Woreda Health Office, Southern Ethiopia Region)

### Time spent on other activities

Activities not related to HEP packages included registration of HHs to community-based health insurance (CBHI) schemes and premium collection, and other emergent activities assigned by the Woreda Health Office or supervising HC, and serving visitors or guests, including health researchers. HEWs frequently reported that Woreda Health Office-level experts are expected to execute and coordinate CBHI. In practice, however, these were considered the most challenging and time-consuming activities for HEWs. They were highly prioritized activities by the Woreda Health Office and given significant weight in HEWs’ performance evaluations. Various participant groups shared these views besides HEWs, including community leaders (e.g. WDA leaders and religious leaders) and community members. Other participants, such as those from HCs and Woreda Health Offices had contrasting views. For them, CBHI activities do not consume HEWs’ time mainly due to the seasonality of such tasks, i.e. on an annual basis.

Being a cabinet member, at least one HEW per HP also participated in political activities at the Kebele level. As part of this engagement, they primarily contributed to other sectors such as education (e.g. dropout tracing), agriculture and rural development (e.g. promoting natural resource conservation, and income tax collection), and social work. In most cases, such tasks tended to receive much attention over the HEP packages due to their urgent nature. Both the HEWs and their supervisors underscored the importance of these activities in influencing the productivity of HEWs.

## Factors that influence work efficiency and time use

As shown in Table 2, the factors that influence HEWs’ time use were categorized under three themes: community context, health system leadership and governance, and HEWs’ job attendance. Each of these is detailed below in terms of its emergent themes.

### Community context

#### Environmental and demographic factors

Rural or urban location of HPs was a major determinant of time use. HEWs in rural areas faced longer travel times and greater logistical challenges, which reduced their overall efficiency. Dispersed settlements in pastoralist settings, challenging topographies, and inaccessibility to transportation services were some of the factors that affected HEWs’ time use, particularly in some difficult times such as rainy seasons. Most pastoralist settings were also too hot and HEWs were forced to work only in the morning shift. These factors led to increased HEWs’ absenteeism and late job attendance. Furthermore, security issues caused sporadic availability of HP staff. Some HPs in pastoralist settings were reportedly unsafe and insecure places to work. As experienced by some participants, snakes and other biting insects lived in such HPs causing protracted absenteeism of HEWs, for example. In urban settings, HEWs could serve more households in a shorter time frame, allowing for more frequent and intensive community engagements.

With regards to demography, the high population size in HP’s catchment area served by fewer numbers and a mix of staff determined the time use and efficiency of HEP services. Most HEWs unanimously described that the actual number of HHs assigned to them was much higher than the standard. They argued that the actual HH size per HEW could be as high as 800 compared to the standard of 300–330 HHs per HEW on average. To effectively use their time, HEWs often had to balance a high service volume with administrative responsibilities, e.g. preparing regular reports.

#### Community trust and engagement

Communities expected HPs to remain open all the time to provide them with clinical services when they need them. As a result, the closure of HPs led to communities’ lack of trust in HEWs. On the other hand, HEWs felt that community engagement activities are crucial for building trust and encouraging participation in health programs. HEWs who spent more time on community-based activities reported stronger relationships with community members, leading to better health outcomes. HEWs who established such relationships felt motivated by the respectful, cooperative, and supportive relationships with the communities they serve, which eventually made their HEP implementation efforts much easier. However, such engagements and efficiency of HEWs were reported to be constrained by certain socio-cultural and economic conditions of communities. For example, beneficiaries were often unavailable on market and worship days for facility and/or outreach-based services, even during emergency situations such as disease outbreaks. It was noted that this would ultimately lead to community noncompliance and de-motivation of HEWs.

#### Local administration

Local administrative decisions and actions influenced HEWs’ time use both positively and negatively. They supported HEWs towards meaningful community engagement for HEP implementation. In contrast, they competed for HEWs’ time and overloaded them with additional non-health and political activities. In most cases, the nature of such tasks was felt as unplanned and urgent. Sometimes, Kebele leaders covered up and favored HEWs’ absenteeism or late attendance for which establishing a strong human resource (HR) management system for HEWs was suggested as a solution.

### Health system leadership and governance

HEWs commonly complained of autocratic, fault-finding, non-humane leadership approaches, and non-supportive supervisory relationships. HEWs experienced insults, humiliation, and a lack of respect from their closest supervisors. They also reported a lack of accountability of health system leadership which overburdened them with a lot of non-HEP and unplanned tasks consuming much of their uncompensated work time. HEWs also felt that they shouldered agonizing accountability to accomplish those non-HEP tasks without proper compensation and for low salaries. For example, when asked about the level of difficulty of tasks, a respondent said:

CBHI is the most difficult task for HEWs to perform. One household head will be registered in 3 forms, and if you miss a code, the whole format will be discarded and you will be forced to re-start the register process. We are given the CBHI task as if it were the 19th HEP package, while it is not. We have never been trained about it, and it doesn’t have performance indicator(s) at the HP level. Yet, it disrupts our work in most cases. Therefore, it needs one additional staff at the HP level, perhaps from four experts who are working in the Woreda CBHI Office. (KII, Woreda Health Office Head, Amhara Region)

Factors related to HR development and management, multisectoral collaboration, and work environment were the commonest sub-themes that emerged under health system leadership and governance theme. These are described below.

#### HR development and management

Inadequate HP staffing both in number and mix despite the extensive HEP work packages was the common HR-related challenge. While some explained the reason as non-compliance to the existing staffing standard (HEW to population ratio); others agreed on the weakness of the existing standard itself as non-accommodative of multiple and complex factors overburdening the HEWs. HPs were reported to be closed oftentimes due to staff shortages. Deploying only female HEWs except in agrarian communities aggravated the shortage, due mainly to frequent maternity leaves for which no gap-filling mechanism existed to ensure continuity of care. Most HEWs considered maternity leave as a strategy for getting rest from their extensive and tiresome work. Thus, considering male HEWs in such settings was perceived to reduce the effect of such leave on care continuity.

Although HEWs claimed to have clear plans and task prioritization skills for facility-based and outreach activities, local health system leaders had a contrasting view. They reported that HEWs lacked planning skills, mostly spending their time on Kebele meetings, and activities not related to HEP packages, or health in general. Some HEWs lacked career development opportunities including training, promotion, and recognition. The level of training and ongoing capacity-building opportunities affected how well HEWs could perform their duties. Some argued that HEWs with more comprehensive training were better equipped to manage time, prioritize tasks, and deliver higher-quality services. Regions that invested in regular training and workshops for HEWs observed better time management. Furthermore, lack of critical appraisal of workload and time required by HEWs to accomplish HEP activities, emergent non-HEP or non-health related activities; and absence of a mechanism to compensate for overload or overtime work were believed to trigger HEWs’ inefficiencies.

#### Multisectoral collaboration

HEWs recognized the positive effect of multisectoral collaboration for efficient and effective social change through HEP implementation. They perceived that it enhances community participation and engagement in outreach activities and campaigns. Nonetheless, it was also acknowledged that multisectoral collaboration challenged HEP implementation. Examples of specific challenges included extensive unplanned administrative or governance tasks to be executed by HEWs, inadequate health workforce to take up integration tasks without compromising sector-specific activities, e.g. HEP, and lack of co-planning where every sector plans for its own making execution more complex.

#### Work environment

Availability of resources such as transportation (bicycles and motorbikes) and communication tools (e.g. mobile phones) was perceived to influence HEWs’ ability to manage their time effectively. HEWs expected a secure residence, working in a relatively developed area, e.g. accessible to transportation, and health insurance services. Yet, these facilities were inadequate in most cases. In addition, the busy work situations of HEWs were reportedly not well understood by administrative and immediate supervisory bodies leading to non-empathic relationships. Some of these challenges were experienced by a respondent as described below:

All HEWs in this woreda don’t reside in houses constructed for them near HP, as they all are living in the nearby City, Butajira. This is a big problem for their time use. They use locally available public transportation facilities like Bajaj and Taxi at their own expense. For example, Degagot Kebele is very far away from the City, incurring up to 200 Birr (≈ $3.6) for a round trip. Given this cost which their salary can’t cover, it is difficult to expect them to go to HP every day. We don’t have a conducive environment to go to each HP daily, and control HEWs’ performance and time use. (KII, HC Head, SNNP Region)

### Job attendance of HEWs

Absenteeism and reporting for work were generally common across settings. Better access to transportation facilities facilitated residential houses for HEWs at or near the HP, availability of required resources and amenities at HPs, absence of security threats, and HEWs’ commitments to professionalism and professional integrity enhanced their availability on the job 5 days a week, i.e. Monday through Friday. For example, one of the participants related HEWs’ absenteeism with unsatisfactory work environment as follows:

Three and four years back, the service [HEP] was very good and we were benefiting much from it. Currently, they [HEWs] are not working here in the community. Of course, there are challenges they face. For example, the HP doesn’t have adequate space due to poor design and construction, which is almost collapsing now. There is no house near the HP for them to live in. As a result, they are not coming to work. (FGD with Women, Harari Region)

The major factors for HEWs’ absenteeism or late job attendance were broadly categorized into three: gender intersectionality, long travel time from home to workplace worsened by limited access to transportation service, and lack of motivation.

#### Gender intersectionality of HEWs

The fact that HEWs are supposed to be females, mainly in agrarian settings, was reported to cause absenteeism or late job attendance due to their gender roles, e.g. maternity leave. Married female HEWs who lived in towns (far from the HP) often spent some time on household chores and caring for family, leading to absenteeism or late job attendance. This was more pronounced on market and worship days, for example.

#### Long travel time from home to workplace and limited access to transportation service

Residence of HEWs far from the HP, and lack of transportation service or unaffordability of the costs were the top-ranking reasons for late job attendance or absenteeism. It was evident that those HEWs living closer to the HP had proper and efficient time use compared to those who lived far away. For example, a HEP supervisor supported the argument as follows:

Experienced HEWs who live close to the HP and far from town use their time effectively. They establish good relations with communities and adhere to their cultures. They start work early in the morning and leave late in the afternoon to serve the community. The community also has no other options. (KII, HEP Supervisor, Oromia Region)

However, HEWs commonly preferred to live in semi-urban areas or towns, relatively far from their workplace, and often failed to access transportation services for various reasons, including poor road infrastructures, unfavorable environmental conditions, e.g. rainy season, and unaffordability of transportation costs when available. Late attendance due to long travel time to the workplace compromised HEWs’ time on the job. For example, some HEWs reported that they arrived at HP at 10:30 AM, 2 hours later than expected.

HEWs had various reasons for preferring to live in semi-urban or urban areas including an established social life there, the need for better access to social services for self and family, e.g. better schools for children, and the absence of safe and secured residence within or near the HP. For example, some HPs in pastoralist settings were reportedly unsafe and insecure, and hence unattended by HEWs for extended periods of time, and became a normative practice. This changed their practice through self-employment as a private healthcare provider, or engagement in private businesses as additional jobs. One respondent described this as:

She [the HEW] works part-time on her own because the HP doesn’t have medicine, so she brings her own medicine. She always comes to the HP but she is scared because there are snakes and dragons inside the HP. Two days ago, the dragon came to her in the HP, and security guards shot it to death. It is still a high-risk area for recurrence of the problem. So, she became frustrated to stay and work in the HP. (FGD of Youth Group-Female, Afar Region)

The study also showed that HEWs could be available in their workplace, but might not be productive. Participants commonly felt that HEWs often come on time to work, but stay idle for various reasons, including lack of drugs and supplies and non-responsiveness to clients’ needs. These were argued as follows, for example:

HEWs spend most of their time in the HP; they are always available in the morning and afternoon. They spend their time sitting in HP because the government does not bring them enough medicine in most cases. Even though we do not get the services we need, they just sit there and spend time. (P8, FGD of Youth Group-Female, Afar Region)

They [HEWs] are physically available at the HP, but they don’t positively respond to patients. When they see a woman waiting for their service, instead of providing services timely, they sit in a room, side-talk there, laugh, eat their lunch there, etc. Then, they may respond to other patients who came late, leaving me to wait for their service in vain. After serving some, they may tell you that they can’t continue providing service and appoint you for another day. Compared to those who get service from them, those who don’t are large in number. The reason for this is inconsiderateness to people’s concerns. (R5, FGD with WDA, Oromia Region)

#### Lack of motivation

Most of the factors described earlier resulted in a lack of motivation among HEWs and affected their commitment and time use. Inadequate salary, unfavorable work environment, increasing work overload mainly imposed by the local administration for which no compensation scheme existed, and lack of health insurance commonly de-motivated HEWs. The fact that HEWs often had maternity leave for a shorter period than their legal entitlement and that they were often denied annual leave were also common.

Economic factors appeared to be the most common reasons for lack of motivation and job dissatisfaction. A low salary was the major one. Some HEWs didn’t get an appropriate salary increment even after upgrading their academic status. A few illustrative expressions of the effect of low salary on HEWs’ motivation are provided below:

The main demotivating reason is economic constraint. Unless they have additional income, they can’t survive with the salary alone. Because of the economic deprivation, they are becoming reckless at work. The current inflation of market prices has made some staff feel hopeless in their work. (KII, HC Head, Oromia Region)

Many health workers are demotivated and have intentions to leave their current job due to low salaries that cannot cover their basic needs. For instance, someone whose family lives in Awbare Woreda center can’t afford to support them with that little salary, and she/he looks for a better job. Therefore, since the salary doesn’t cover costs related to one’s living and family support, they prefer leaving the job. (KII, HP Head, Somali Region)

Participants were also asked what actions would help motivate HEWs. Accordingly, communities’ satisfaction with the HEP services they provide, and the respect and support they provide for HEWs were the common reasons for HEWs’ motivation which helped them reduce burnout and attrition. Particularly, treating and healing mothers and children satisfied them, and those working in comprehensive HPs were motivated by their achievements in the recently introduced HEP optimization initiative where additional clinical services, including essential laboratory tests, are offered. Although rarely accessible, HEWs also recognized the motivational role of increased salaries and compensation, and career development opportunities, e.g. education, promotion, and recognition.

## Discussion

HEWs divided their time between facility- and outreach-based service delivery modalities, at a ratio of 2:3 days per week, and spent much of their time providing maternal and child health (MCH) and HES packages of HEP. As would be expected, services delivered at outreach sites, including campaigns and door-to-door, were more time-consuming than facility-based services. The results also revealed that other activities which were neither related to the HEP packages, nor to the healthcare in general competed for a considerable portion of HEWs’ time. Examples included their roles in CBHI implementation, agricultural and rural development and education sectors, and other political assignments. Factors related to community context, health system leadership and governance, and availability of HEWs on their job influenced HEWs’ work efficiency and time use. Environmental and demographic attributes, community trust and engagement, and local administration belonged to the community context. Aspects of health system leadership and governance were HR development and management, multisectoral collaboration, and work environment; while job attendance of HEWs was characterized by gender intersectionality, long travel time from home to workplace limited access to transportation service, and lack of motivation. Overall, these findings are consistent with many previous studies [5,8,12,17–35]. For example, Fekadu et al. [25] cited the significant impact of CBHI activities on HEWs’ workload, and Zerfu et al. [18] noted the role of contextual and health system-related challenges including integration and collaboration with other sectors, logistics, and adequacy and appropriateness of the number and gender mix of service providers.

The findings on-time distribution between facility and outreach-based services and the emphasis given to the MCH and HES packages align with the recommended practices of HEP [5]. The perceived consequences of non-productive tasks on HEWs’ efficiency observed in this study are also supported by the literature [20–29,36,37]. The influence of environmental and demographic factors such as scattered settlements, challenging topographies aggravated by inaccessibility to transportation services, security concerns, and mismatch between the number of HEWs and population size to be served on HEWs’ time use is also well documented [18,36,37]. For example, a study [18] cited not updating the staffing profile of HEP despite the dynamics of demand for health services and population growth as a limitation of the program. The fact that an HP serves an average of 6,875 people, far exceeding the recommended 3,000–5,000, has also been reported [36,37]. Population growth, expansion of Kebeles, rugged topography and geographic setups of rural areas, and poor access to all-weather roads have been the critical challenges to handling HEP activities by two female HEWs [18].

Failure to meet community expectations led to communities’ lack of trust in HEWs, resulting in inefficiency, while HEWs who established strong relationships with communities were encouraged by respectful, cooperative, and supportive relationships. This is also documented in the literature [7,8,18]. Communities may lose trust in HEWs for various reasons such as poor quality of care which needs further investigation. Similar to previous studies [5,7,8,12,18,35], the study revealed that HEWs commonly experienced unfavorable leadership practices, non-supportive relationships with their immediate supervisors, and the absence of an accountability system for workload analysis and compensation mechanism for work overload, resulting in their inefficiencies. Inadequate HP staffing both in number and mix despite the extensive HEP work packages was the common HR-related challenge. HEWs also lacked planning skills, and career development opportunities including training, promotion, and recognition.

Although HEWs recognized the contributions of multisectoral collaboration for the delivery of HEP services through community engagement, they also noted the disadvantages such as extensive unplanned administrative or governance tasks which were unrelated to health. The work environment was mostly inconvenient due to a lack of resources such as a secure residence, transportation, and communication tools. This mostly influenced HEWs’ ability to manage their time effectively. HEWs often had maternity leave for a shorter period than their legal entitlement and were often denied annual leave. These have also been documented in other studies [5,8,12,18]. For example, Mangham-Jefferies, et al. [12] noted that a quarter of HEWs were involved in land use tax and agricultural income tax collection, and 12% had been on maternity leave. Dual responsibility of HEWs, weak intersectoral collaboration, and weak community involvement in the governance of HEP were also considered major governance issues of HEP [8]. Imposing multiple responsibilities on HEWs with tasks that are not related to health is likely to create competing priorities for HEWs and affect their time use [10,21,38–41]. This suggests the need for accommodative planning approaches, and establishing a clear accountability structure for HEP implementation, particularly at the Kebele level.

Absenteeism and reporting for work were generally common across settings. Better access to transportation facilities facilitated residential houses for HEWs at or near the HP, availability of required resources and amenities at HPs, absence of security threats, and HEWs’ commitments to professionalism and professional integrity enhanced their availability on the job 5 days a week. Gender intersectionality (being female HEWs mainly in agrarian settings), long travel time from home to workplace worsened by limited access to transportation service, and lack of motivation were the major reasons for HEWs’ absenteeism or late job attendance.

Hopkins [42] defines intersectionality as a way of understanding social relations by examining intersecting forms of discrimination and addressing all potential roadblocks to an individual or group’s well-being. Intersectionality can also consider the privileges or advantages that people experience in line with their social identities, and how those advantages and disadvantages interact and overlap [42]. Applying the concept to the HEP strategy, while addressing gender equity through affirmative actions by creating better employment opportunities for rural women as HEWs, and assuming that female community health workers can better identify and manage maternal and child health problems than males; the fact that female HEWs are confronted by several hardships related to working in rural settings implies gender intersectionality among most HEWs. Besides, the gender roles they assumed in society caused absenteeism or late job attendance in most cases. This finding favors the recently developed HEP optimization roadmap [7] where involving male HEWs has been planned as part of the reform. Other studies similarly identified the gap, e.g. Zerfu et al. [18].

Despite the advantages of having HEWs residing near the HP, most HEWs lived outside their Kebele where the HP is located. This is consistent with what has been reported in Mangham-Jefferies, et al. [12]. This results in long travel time and late job attendance. The study also showed that HEWs could be available in their workplace, but might not be productive. This may partly be due to communities bypassing HPs and seeking care directly from HCs. The expansion of HCs to rural areas has created a new combination of health facilities where both an HC and an HP may be located within the same rural Kebele, and communities may bypass HPs, leaving HEWs idle [8]. Inaccessibility of services for communities due to reasons such as health-seeking behavior, road access, difficult topography, long distances, and lack of transportation could also be part of the explanation [5,8].

Communities’ satisfaction with the HEP services they provide, and the respect and support they provide for HEWs were the common reasons for HEWs’ motivation which helped them reduce burnout and attrition. In contrast, low salaries, unfavorable work environment, increasing work overload mainly imposed by the local administration for which no compensation scheme existed, and lack of health insurance commonly de-motivated HEWs, and affected their commitment and time use. These have also been reported previously [8,31,35]. For example, Ejigu et al. [31] cited that psychosocial factors, administrative issues, career advancement incentives, and workplace-related problems were the reasons for the attrition of HEWs. The study also showed that the lack of official recognition and the long distance between the district health office and HP increased the likelihood of HEWs’ attrition.

The strength of this study is that it attempted to capture the views of multiple actors of HEP implementation including HEWs, their supervisors, and community leaders and members. The perspectives of various HEP implementation approaches (i.e. rural, agrarian, and pastoralist settings), and seasonal task variability have also been represented. However, the results reported in this paper should be interpreted with caution as they relied on perceptions or subjective responses. In addition, all COREQ points are not completely covered, e.g. information about personal characteristics of the research team in terms of gender, and whether repeat interviews were carried out, were missing.

## Conclusion

HEWs commonly spend their time on productive non-health or non-HEP activities such as those related to community-based health insurance, agricultural and rural development and education sectors, and other political assignments were perceived as significant. The study also showed that HEWs could be available in their workplace, but might not be productive. Common factors for the inefficiencies across HPs pertained to community context, health system leadership and governance, and underlying causes of absenteeism. Environmental and demographic attributes, community trust and engagement, and local administration belonged to the community context; and aspects of health system leadership and governance were HR development and management practices, multisectoral collaboration, and work environment. HEWs’ job attendance was constrained by gender intersectionality of HEWs, long travel time, limited access to transportation service, and lack of motivation – which also results from other factors.

Therefore, strategies need to be devised to address inefficiencies resulting from the identified modifiable factors. These include improving accessibility to transportation services, security conditions, and matching the workforce with population size. Management, leadership, and governance practices of the district health system should also be enhanced, e.g. through introducing accommodative planning approaches to multisectoral collaboration and community engagement, and establishing a clear accountability structure for HEP implementation at the *Kebele* level. Capacity building through need-based training, and introducing or strengthening incentive mechanisms is essential to keep HEWs motivated and capable. Specifically, absenteeism could be remedied through creating conducive work environment, introducing incentive mechanisms, and establishing accountability system.

## Supplementary Material

Supplementary File 1_COREQ 32 Item Checklist_Completed.docx
